# Association between serum uric acid levels and long-term mortality of metabolic dysfunction-associated fatty liver disease: a nationwide cohort study

**DOI:** 10.1186/s13098-023-00997-z

**Published:** 2023-02-23

**Authors:** Zhening Liu, Qinqiu Wang, Hangkai Huang, Xinyu Wang, Chengfu Xu

**Affiliations:** 1 Department of Gastroenterology, The First Affiliated Hospital, Zhejiang University School of Medicine, 79 Qingchun Road, Hangzhou, 310003 China; 2 Zhejiang Provincial Clinical Research Center for Digestive Diseases, Hangzhou, 310003 China

**Keywords:** Uric acid, Fatty liver disease, Metabolic disorders, Mortality, Non-linearity

## Abstract

**Background:**

The association between hyperuricemia and metabolic dysfunction-associated fatty liver disease (MAFLD) remains undetermined. This study aimed to examine the association of serum uric acid (SUA) levels with prevalence and long-term mortality of MAFLD in a nationally representative sample of US adults.

**Methods:**

This analysis included 11,177 participants from the Third National Health and Nutrition Examination Survey (NHANES III, 1988–1994) with matched mortality data until 2019. We used logistic regression models to estimate the adjusted odd ratios (ORs) for factors associated with risk of MAFLD, and applied restricted cubic spline (RCS) regression to assess the non-linear associations of SUA levels with all-cause and cause-specific mortality of MAFLD. We also used Cox proportional hazards regression analysis to estimate hazard ratios (HRs) for the mortality.

**Results:**

A higher SUA level contributed to a significant increased risk of MAFLD. every 1 mg/dL increment of SUA level was related to 17% (95% CI 9–24%) increased risk of MAFLD. Furthermore, a U-shaped association for males and a J-shaped association for females was discovered between SUA levels and all-cause mortality in participants with MAFLD. Specifically, among males, when SUA > 6.7 mg/dL, the higher SUA showed increased risk of cardio-cerebrovascular disease (CVD) mortality [HR (95% CI): 1.29 (1.05–1.58)]. As for females, only when SUA > 5.5 mg/dL, it showed a significantly positive association with risk of CVD and cancer mortality [HR (95% CI) 1.62 (1.24–2.13) and 1.95 (1.41–2.68)].

**Conclusions:**

Elevated SUA level is significantly associated with an increased risk of MAFLD. Besides, SUA level is also a predictor of long-term mortality of MAFLD.

**Supplementary Information:**

The online version contains supplementary material available at 10.1186/s13098-023-00997-z.

## Introduction

Metabolic dysfunction-associated fatty liver disease (MAFLD) is a consensus-driven concept with the prevalence estimated to be 34.8% in the contemporary US population [1, 2]. MAFLD emphasizes the importance of metabolic dysfunction complicated with fatty liver, and is more inclusive in its etiology than non-alcoholic fatty liver disease (NAFLD) [3]. Moreover, compared with NAFLD, MAFLD patients had an increased risk of fibrosis [4], all-cause and cardiovascular mortality [5], indicating a significant group of people with more comorbidities and worse prognosis [6].

The core characteristic of MAFLD is metabolic dysfunction [3], which was closely associated with hyperuricemia. The prevalence of metabolic syndrome increases substantially with increasing levels of SUA among Chinese [7], Japanese [8], and the US population [9]. Additionally, a meta-analysis by Kodama et al. revealed that SUA level was positively associated with the development of type 2 diabetes regardless of various study characteristics [10]. We previously observed that SUA level was positively associated with the prevalence of NAFLD [11]. Furthermore, in NAFLD patients, hyperuricemia was independently associated with the severity of liver damage [12] and liver fibrosis [13]. Prospective studies also showed that hyperuricemia significantly preceded NAFLD [14, 15]. However, there is lack of evidence to evaluate the association between SUA and MAFLD.

MAFLD has substantial heterogeneity and may demonstrate different mortality outcome based on its subtypes [1]. In a Chinese cohort, MAFLD with positive hepatitis B surface antigen or excessive alcohol consumption further increased the risk of death [16]. Furthermore, Chen et al*.* reported that those with lean MAFLD and diabetic MAFLD may have higher risks of all-cause mortality than those with overweight/obese MAFLD [17]. The association between SUA and the risk of mortality varies across studies and differs between men and women [18, 19]. Notably, in specific populations, this association will not be exactly the same either. In patients receiving hemodialysis, higher SUA level was associated with lower risk of all-cause and CVD mortality [20], but Ong et al. reported that SUA was not an independent predictor of all-cause or CVD mortality in community-based type 2 diabetes patients [21]. Nevertheless, whether SUA predicts long-term mortality of MAFLD patients remains unknown.

In view of the above questions, we aimed to explore the association of SUA with prevalence and long-term mortality of MAFLD in a representative sample of the United States population.

## Methods

### Study population

The NHANES III is a stratified, multistage clustered design study conducted by the National Center for Health Statistics (NCHS) from October 1988 through October 1994 in two phases, to access a representative sample of the United States non-institutionalized civilian population. There were 14,797 NHANES III adults aged ≥ 20 years subsampled to fast before attending a morning exam session. We excluded those with missing essential laboratory data (*n* = 3620), and the final subpopulation sample included for this analysis consisted of 11,177 participants. Of these, 3376 participants were diagnosed MAFLD and available for the survival analyses (Additional file 1: Fig. S1). The survey was approved by the Institutional Review Board of the Centers for Disease Control and Prevention. All participants signed informed consent.

### Measurements and key variables

Hepatic steatosis was recorded by ultrasound (Toshiba Sonolayer SSA-90A) and evaluated using the following five parameters (liver to kidney contrast, parenchymal brightness, bright vessel walls, deep beam attenuation, and gallbladder wall definition) by three trained ultrasound readers. The degree of hepatic steatosis was graded as normal, mild, moderate, or severe.

MAFLD was defined as the presence of hepatic steatosis (mild to severe) with one or more of the following: (i) overweight or obese (body mass index ≥ 25 kg/m^2^); (ii) type 2 diabetes (fasting plasma glucose ≥ 126 mg/dL or HbA1c ≥ 6.5% or the use of anti-hyperglycemic agents); or (iii) at least 2 metabolic abnormalities described by any two indicators: (a) waist circumference (WC) ≥ 102 cm in men or ≥ 88 cm in women; (b) blood pressure ≥ 130/85 mmHg or taking anti-hypertension drugs; (c) raised triglycerides (≥ 1.70 mmol/L); (d) reduced HDL cholesterol (plasma HDL < 1.0 mmol/L for men and < 1.3 mmol/L for women); (e) prediabetes status (FPG 5.6–6.9 mmol/L, or 2-h post-load glucose levels 7.8–11.0 mmol or HbA1c 5.7–6.4%); (f) HOMA-IR ≥ 2.5; (g) plasma C-reactive protein (CRP) level > 2 mg/L [3].

Blood samples for SUA measurement were measured by uricase-mediated oxidation to form allantoin and hydrogen peroxide (Hitachi 737 Analyzer), and data were rounded 1 decimal places (0.1 mg/dL) [22].

### Covariates

For participants who had smoked at least 100 cigarettes during their lives, those smoked at the time of the interview were classified as current smokers, while those who did not currently smoke were former smokers. Otherwise, they were classified as never smokers. Alcohol drinkers were classified as people who consumed at least 12 drinks in their entire life and had > 3 drinks/day for men or > 2 drinks/day for women in the past 12 months. Sedentary behavior was defined if participants didn’t do the following activities in the past month: jogging/running, bicycling, swimming, aerobics, other dancing, calisthenics, garden/yard work, weight lifting, or other sports. The estimated glomerular filtration rate (eGFR) was calculated using the simplified modification of diet in renal disease (MDRD) formula [23]:

$$\begin{aligned} {\text{eGFR}}\left( {{\text{mL}}/\min /1.73\;{\text{m}}^{2} } \right) & = 175 \times \left( {{\text{serum}}\;{\text{creatinine}}} \right)^{ - 1.154} \hfill \\ & \quad \times \left( {{\text{age}}} \right)^{ - 0.203} \times \left( {0.742\;{\text{if}}\;{\text{female}}} \right) \hfill \\ \end{aligned}$$

### Follow-up and death ascertainment

All participants over 20 years were followed for mortality until December 31, 2019. Vital status and cause of death assignment were based on the National Death Index (NDI) death certificate records. The leading causes of death (UCOD_LEADING) was defined by the International Classification of Diseases coding (ICD-10) and used for case ascertainment. Specifically, CVD mortality (ICD-10 codes I00-I09, I11, I13, I20-I69) included all deaths from ischemic heart disease, cerebrovascular disease, and other atherosclerotic heart diseases, while cancer mortality included deaths from malignant neoplasm (ICD-10 codes C00–C97).

### Statistical analysis

To compare baseline characteristics, we used Surveymeans and Surveyfreq procedures to describe variables in weighted forms. Surveyreg procedure for continuous variables and Rao-Scott Chi-square test for categorical variables were used to test the statistical difference. Then, multivariate logistic regression models with increasing degrees of adjustment were employed to assess associations between SUA and MAFLD. Model 1 was adjusted for demographic factors: age, sex, race/ethnicity (non-Hispanic white, non-Hispanic black, Mexican–American, or others), marital status (never, married, or others), education level (below high school, high school, or over high school), and occupation type (work at a job or business, or others). Model 2 was adjusted for model 1 plus lifestyle factors: smoking status (never, former, or current), alcohol consumption (active, or inactive), physical exercise (sedentary or active), BMI, and eGFR. Model 3 was further adjusted for model 2 plus biochemistry factors: triglyceride, total cholesterol, fasting glucose, CRP, and ALT. Furthermore, considering SUA has a sex-specific influence on mortality reported previously, survival analysis was conducted stratified by sex among participants with MAFLD.

We plotted Kaplan–Meier curves to compare the all-cause, CVD-specific, and cancer-specific mortality of different SUA groups (log-rank test). Then, to address the potential nonlinear association between SUA and survival outcomes, restricted cubic splines with knots at the 5th, 35th, 65th, and 95th percentiles of SUA distribution were produced by SAS macro *% RCS_Reg* [24]. Based on the threshold identified by RCS, 2-piecewise Cox proportional hazards models were developed to estimate the hazard ratio (HR) and 95% confidence interval (CI) of SUA with mortality, after adjusting for potential confounders. SAS 9.4 (SAS Institute Inc., Cary, NC) was employed for all analyses in this study. Two-sided *P* < 0.05 was considered statistical significance.

## Results

### Baseline characteristics

A total of 11,177 participants (5264 men and 5913 women) with a mean age of 41.85 years were included in baseline analysis. The baseline characteristics of participants in each SUA group are summarized in Table 1. Compared with participants with SUA in the lowest group (< 4.0 mg/dL), those with higher SUA levels were older, male predominant, less educated, had higher BMI, waist circumference, systolic and diastolic blood pressure, and higher serum alanine aminotransferase (ALT), fasting triglyceride, total cholesterol, blood glucose, insulin and C-reactive protein levels, but lower serum HDL-C levels (Table 1).

**Table 1:** Baseline characteristics of participants according to SUA levels

Variables	Total (*n* = 11,177)	Serum uric acid (mg/dL)					
		Group 1 (< 4.0)	Group 2 (< 4.0–4.9)	Group 3 (5.0–5.9)	Group 4 (6.0–6.9)	Group 5 (≥ 7.0)	*P*
Age, year	41.85 ± 0.37	39.38 ± 0.43	41.07 ± 0.44	42.49 ± 0.61	42.80 ± 0.54	44.45 ± 0.6	< 0.001
Male, %	49.2 (0.5)	9.1 (1.2)	30.7 (1.1)	59.4 (1.5)	78.5 (1.5)	82.7 (1.7)	< 0.001
Race/ethnicity, %							0.261
Non-Hispanic White	76.4 (1.4)	77.4 (2.0)	76.3 (1.6)	76.6 (1.6)	75.9 (1.6)	75.0 (2.0)	
Non-Hispanic Black	10.4 (0.6)	10.1 (0.9)	10.0 (0.7)	10.5 (0.7)	9.8 (0.8)	12.5 (1.1)	
Mexican American	5.4 (0.5)	5.7 (0.5)	5.4 (0.5)	5.4 (0.5)	5.4 (0.7)	5.1 (0.5)	
Marital status, %							0.007
Never married	17.3 (0.8)	18.1 (1.6)	16.3 (1.1)	17.7 (1.4)	20.2 (1.9)	12.8 (1.4)	
Married	63.2 (0.9)	22.3 (1.6)	21.4 (1.1)	19.9 (1.3)	15.6 (1.3)	16.2 (1.3)	
Others	19.5 (0.7)	59.5 (1.7)	62.3 (1.7)	62.3 (1.3)	64.2 (1.8)	70.9 (1.7)	
Education level							0.029
Below high school	22.4 (1.0)	19.2 (1.8)	21.4 (1.4)	22.8 (1.3)	24.7 (1.7)	24.9 (2.1)	
High school	34.6 (0.8)	37.9 (1.5)	34.5 (1.5)	33.0 (2.1)	32.7 (1.4)	32.7 (1.4)	
Over high school	43.0 (1.3)	40.7 (1.6)	42.7 (1.9)	42.3 (2.1)	42.4 (2.3)	42.4 (2.3)	
Work at a job or business, %	71.6 (0.8)	70.2 (1.4)	69.8 (1.4)	72.3 (1.3)	73.9 (1.5)	72.3 (1.4)	0.164
Smoking status, %							< 0.001
Never	44.6 (0.9)	53.3 (1.6)	53.3 (1.6)	46.4 (1.5)	42.5 (1.5)	37.7 (2.4)	
Former	25.4 (0.7)	18.0 (1.4)	22.3 (1.0)	27.0 (1.4)	28.9 (2.1)	34.1 (1.8)	
Active	30.0 (0.9)	28.7 (1.4)	31.2 (1.4)	30.5 (1.4)	30.3 (1.7)	28.2 (2.1)	
Alcohol drinkers, %	20.8 (0.9)	13.3 (1.5)	18.4 (1.3)	21.4 (1.1)	25.2 (2.0)	28.5 (2.1)	< 0.001
Sedentary behavior, %	19.9 (0.9)	21.7 (1.6)	21.3 (1.3)	18.5 (1.4)	19.4 (1.4)	18.4 (1.6)	0.262
Body mass index, kg/m^2^	26.53 ± 0.12	23.91 ± 0.15	25.44 ± 0.11	26.98 ± 0.21	27.95 ± 0.17	29.65 ± 0.24	< 0.001
Waist circumference, cm	91.62 ± 0.26	82.14 ± 0.41	87.75 ± 0.40	93.25 ± 0.42	97.25 ± 0.40	101.99 ± 0.51	< 0.001
Systolic blood pressure, mmHg	122.29 ± 0.38	114.97 ± 0.56	119.57 ± 0.46	123.71 ± 0.50	126.85 ± 0.66	129.07 ± 0.68	< 0.001
Diastolic blood pressure, mmHg	75.31 ± 0.26	71.28 ± 0.35	73.67 ± 0.29	76.12 ± 0.36	77.95 ± 0.41	79.13 ± 0.52	< 0.001
Alanine aminotransferase, U/L	18.03 ± 0.43	13.76 ± 0.51	15.33 ± 0.38	18.09 ± 0.46	22.06 ± 0.77	23.75 ± 0.75	< 0.001
C-reactive protein, mg/dL	0.39 ± 0.01	0.38 ± 0.02	0.38 ± 0.01	0.38 ± 0.02	0.39 ± 0.02	0.47 ± 0.02	0.009
Triglyceride, mmol/L	1.57 ± 0.02	1.10 ± 0.02	1.35 ± 0.02	1.56 ± 0.03	1.83 ± 0.03	2.31 ± 0.11	< 0.001
Cholesterol, mmol/L	5.23 ± 0.02	4.91 ± 0.03	5.15 ± 0.03	5.29 ± 0.03	5.35 ± 0.04	5.52 ± 0.05	< 0.001
HDL-C, mmol/L	1.31 ± 0.01	1.47 ± 0.02	1.39 ± 0.02	1.28 ± 0.01	1.18 ± 0.01	1.13 ± 0.02	< 0.001
Fasting blood glucose, mmol/L	5.40 ± 0.02	5.27 ± 0.08	5.28 ± 0.04	5.42 ± 0.04	5.54 ± 0.05	5.58 ± 0.04	< 0.001
Fasting insulin, μU/mL	10.69 ± 0.23	8.42 ± 0.35	9.15 ± 0.24	10.66 ± 0.33	12.2 ± 0.34	15.01 ± 0.68	< 0.001
HOMA-IR	2.83 ± 0.08	2.20 ± 0.14	2.37 ± 0.09	2.84 ± 0.13	3.26 ± 0.15	4.07 ± 0.25	< 0.001

### Associations of SUA levels with the prevalence of MAFLD

**Fig. 1:**
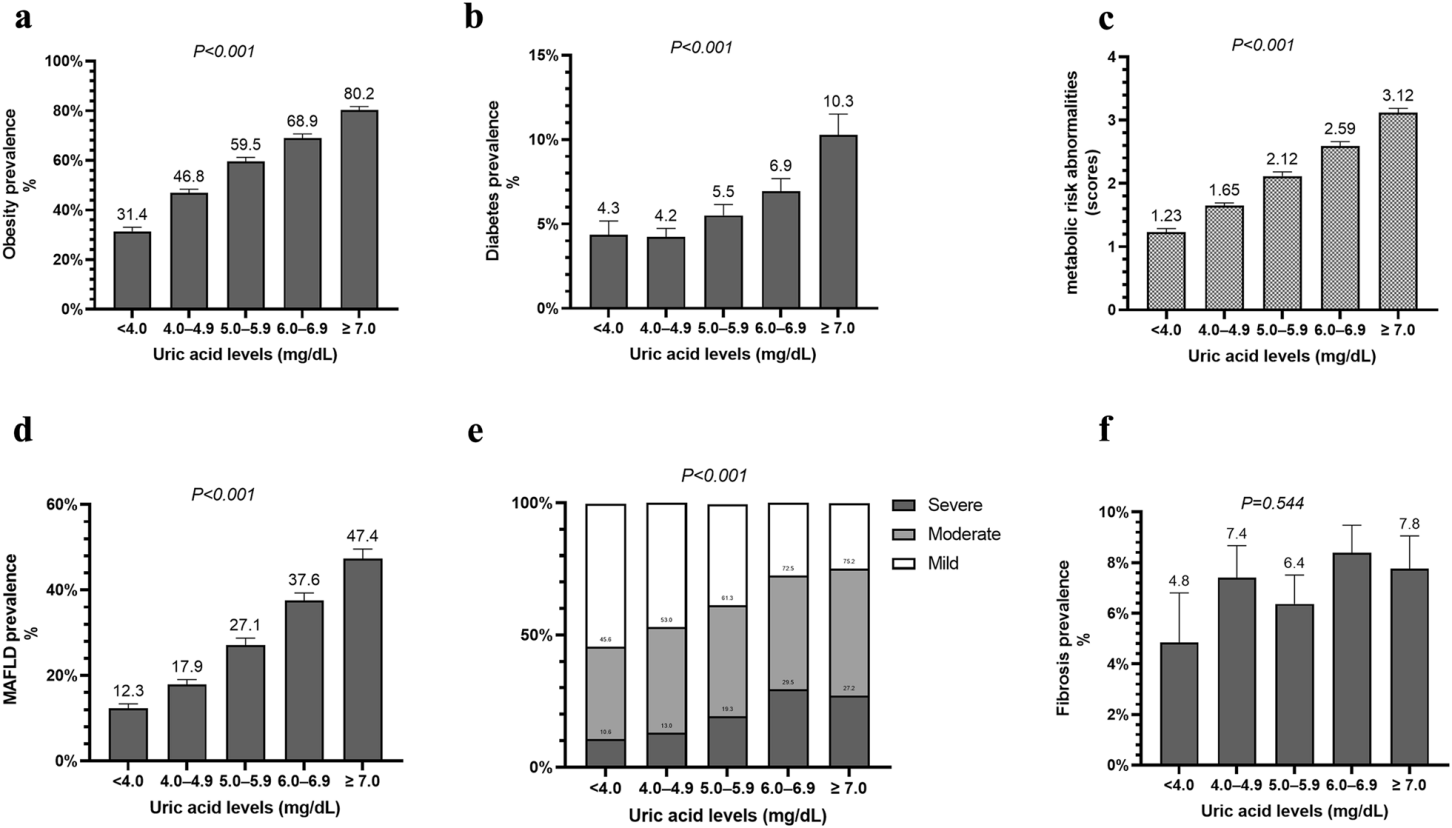
Association between SUA and MAFLD and its related metabolic disorders. **a** Prevalence of obesity, **b** prevalence of diabetes, **c** numbers of metabolic risk abnormalities, **d** prevalence of MAFLD, **e** severity of hepatic steatosis in MAFLD patients, and **f** prevalence of advanced fibrosis in MAFLD patients

As illustrated in Fig. 1, SUA levels were positively associated with the prevalence of obesity, diabetes, and the number of metabolic risk abnormalities (all with *P* < 0.001, Fig. 1a–c). We also found that SUA levels were positively associated with the prevalence of MAFLD, which was almost four times higher in the highest group than that in the lowest one (47.4% vs 12.3%, *P* < 0.001, Fig. 1d). Furthermore, in participants with MAFLD, SUA levels were significantly associated with the severity of hepatic steatosis (*P* < 0.001, Fig. 1e), but not with advanced fibrosis (*P* = 0.544, Fig. 1f). These results indicated a significant association of SUA levels with MAFLD and its related metabolic abnormalities.

### Associations of SUA levels with the risk of MAFLD

We employed complex sample logistic regression analysis with increasing degrees of adjustment to estimate the risk of MAFLD across SUA levels (Table 2). In the minimally adjusted model that included only demographic characteristics (model 1), the ORs (95% CIs) of MAFLD for participants with SUA in groups 2–5 were 1.53 (1.21–1.93), 2.69 (2.06–3.52), 4.56 (3.58–5.82), and 6.72 (5.19–8.70). This positive association remained significant after adjusting for lifestyle factors in model 2, and additional biochemistry factors in model 3 [compared with those in the first group, ORs (95% CIs) of MAFLD for participants with SUA levels in groups 2–5 were 1.10 (0.84–1.45), 1.33 (1.01–1.75), 1.65 (1.27–2.14), and 1.72 (1.25–2.37)]. Furthermore, our multivariable dose–response analysis showed that every 1 mg/dL increment of SUA was related to 17% increased risk of MAFLD [OR_continuous_ (95% CI): 1.17 (1.09–1.24), model 3]. These findings suggested a positive liner association of SUA levels with risk of MAFLD.

**Table 2:** Association of SUA levels with risk of MAFLD

Male	Serum uric acid [OR (95%CI)]					*P* for trend	Continuous^a^
	Group 1 (< 4.0 mg/dL)	Group 2 (4.0–4.9 mg/dL)	Group 3 (5.0–5.9 mg/dL)	Group 4 (6.0–6.9 mg/dL)	Group 5 (≥ 7.0 mg/dL)		
Model 1	1 (ref)	1.53 (1.21–1.93)	2.69 (2.06–3.52)	4.56 (3.58–5.82)	6.72 (5.19–8.70)	< 0.001	1.57 (1.48–1.66)
Model 2	1 (ref)	1.12 (0.86–1.45)	1.44 (1.11–1.86)	2.03 (1.60–2.59)	2.46 (1.86–3.26)	< 0.001	1.27 (1.20–1.35)
Model 3	1 (ref)	1.10 (0.84–1.45)	1.33 (1.01–1.75)	1.65 (1.27–2.14)	1.72 (1.25–2.37)	< 0.001	1.17 (1.09–1.24)

Model 1 was adjusted for demographic factors: age, sex, race/ethnicity, marital status, education level, and occupation type

Model 2 was adjusted for model 1 plus lifestyle factors: smoking status, alcohol consumption, physical exercise, BMI, and eGFR

Model 3 was further adjusted for model 2 plus biochemistry factors: triglyceride, total cholesterol, fasting glucose, CRP, and ALT

^a^OR for 1 mg/dL SUA increase

### Associations of SUA levels with the long-term mortality of MAFLD

The significant association of SUA levels with risk of MAFLD promotes us to further explore whether SUA levels are associated with long-term mortality of MAFLD. Considering SUA levels has a sex-specific influence on mortality, we conducted the sex-stratified analysis among participants with MAFLD. During a median follow-up of 25.8 years (906,930 person-years), 1510 deaths were recorded (including 280 CVD deaths, 191 cancer deaths, and 814 all-cause deaths in males; and 217 CVD deaths, 153 cancer deaths, and 696 all-cause deaths in females).

**Fig. 2:**
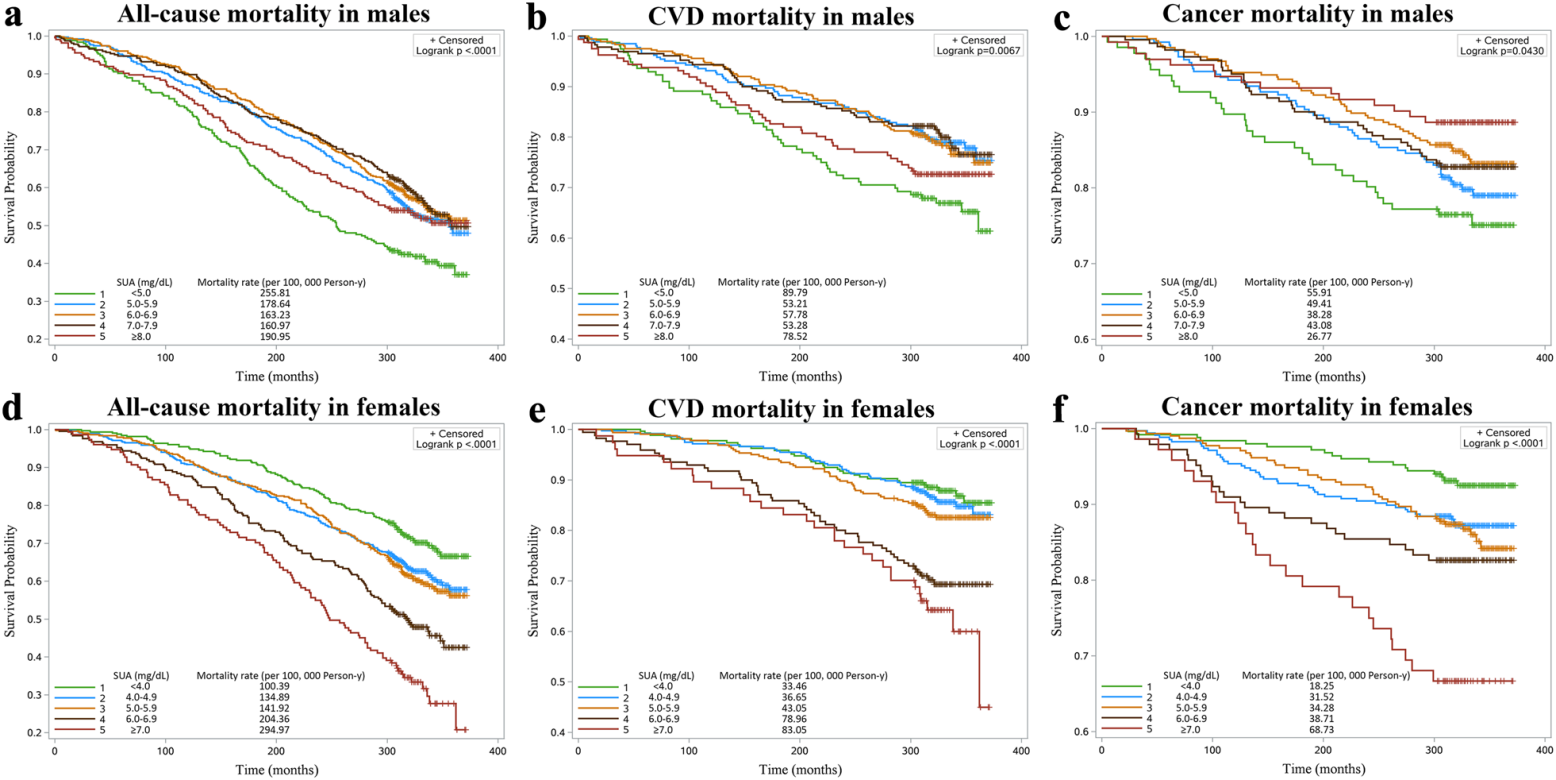
Kaplan–Meier survival estimates according to the groups of uric acid for the probability of all-cause and cause-specific mortality among men (**a**–**c**) and women (**d**–**f**) (*P* < 0.05 for all by log-rank test)

Kaplan–Meier curves of survival showed that in males (Fig. 2a), group 1 (SUA < 5.0 mg/dL) had the highest all-cause mortality (crude mortality rate: 255.81/100,000 person-years), followed by group 5 (SUA ≥ 8.0 mg/dL, crude mortality rate: 190.95/100,000 person-years), nearly appearing a U-shaped relation. Interestingly, in females (Fig. 2d), the crude mortality rate increased monotonically with SUA levels, that is, the lowest one was in group 1 (SUA < 4.0 mg/dL, crude mortality rate: 100.39/100,000 person-years), and the highest one was in group 5 (SUA ≥ 7.0 mg/dL; crude mortality rate: 294.97/100,000 person-years).

Specifically, CVD mortality shared similar pattern with all-cause mortality in both genders (Fig. 2b,e). However, in terms of cancer mortality, only group 1 had a significantly higher crude mortality rate in males (55.91/100,000 person-years, *P* = 0.043; Fig. 2c), while there was a monotonical relation in females (Fig. 2f).

### Nonlinear association and threshold effect analysis between SUA and long-term mortality in participants with MAFLD

**Fig. 3:**
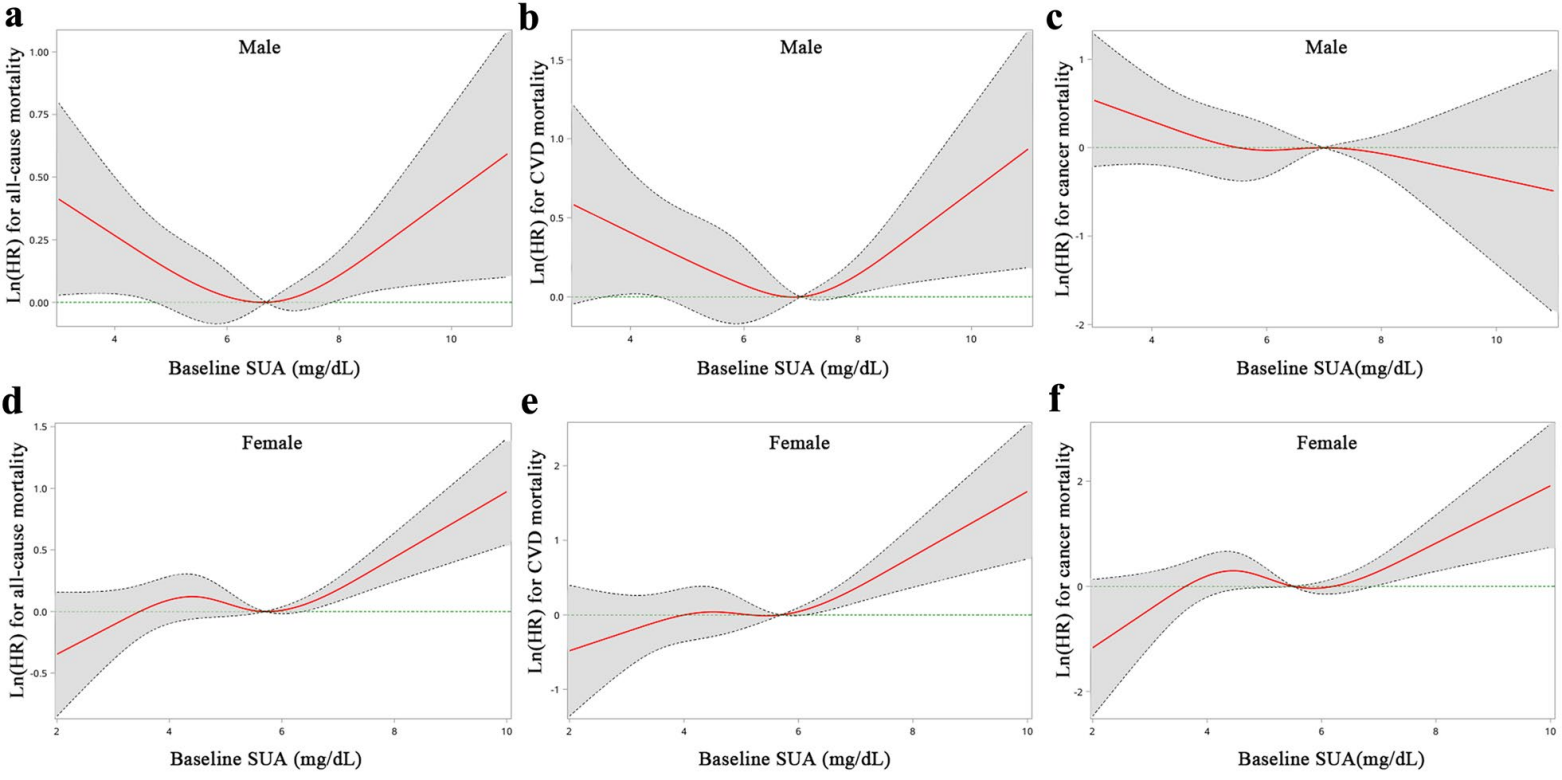
Dose–response relationships between baseline SUA and the Ln(HR)s of all-cause and cause-specific mortality among men (**a**–**c**) and women (**d**–**f**). Red lines represent adjusted hazard ratios [with 95% CI (dashed lines)] based on restricted cubic splines with knots at the 5th, 35th, 65th, and 95th percentiles of uric acid distribution. Model adjusted for age, sex, race/ethnicity, marital status, education level, occupation type, smoking status, alcohol consumption, physical exercise, BMI, eGFR, triglyceride, total cholesterol, fasting glucose, CRP, and ALT

We applied restricted cubic spline regression to assess the nonlinear association of SUA levels with all-cause and cause-specific mortality in participants with MAFLD. As depicted in Fig. 3a, cubic spline models showed a U-shaped association between SUA levels with all-cause mortality among males. This may partly attribute to the J-shaped association with CVD mortality (Fig. 3b) and the monotonically decreasing association with cancer mortality (Fig. 3c). For females, SUA levels showed a J-shaped association with all-cause mortality (Fig. 3d), CVD mortality (Fig. 3e) and cancer mortality (Fig. 3f).

We then fitted the association between SUA and cause-specific mortality using the 2-piecewise Cox proportional hazards model to estimate specific threshold effects (Table 3). Among males, when SUA > 6.7 mg/dL, elevated SUA levels significantly increased risk of CVD mortality [HR (95% CI): 1.29 (1.05–1.58)], and there was a linear trend for cancer mortality [HR (95% CI): 0.80 (0.67–0.96)]. Among females, when SUA > 5.5 mg/dL, elevated SUA levels showed a significantly positive association with risk of CVD mortality [HR (95% CI): 1.62 (1.24–2.13)] and cancer mortality [HR (95% CI): 1.95 (1.41–2.68)]. These results suggested that SUA levels of 6.7 mg/dL for males or 5.5 mg/dL for females may be a threshold for the increased long-term mortality of MAFLD patients.

**Table 3:** Threshold effect analysis of SUA on all-cause, CVD, and cancer mortality

Threshold (mg/dL)	HR (95% CI)		
	Univariate	Age-adjusted	Multivariate^a^
Male			
All-cause mortality			
≤ 6.7	0.77 (0.65–0.92)	0.86 (0.76–0.97)	0.95 (0.80–1.13)
> 6.7	1.38 (1.03–1.85)	1.22 (0.95–1.55)	1.07 (0.90–1.26)
CVD mortality			
≤ 6.7	0.77 (0.60–1.00)	0.89 (0.72–1.11)	1.10 (0.85–1.43)
> 6.7	1.50 (1.13–2.00)	1.33 (1.10–1.60)	1.29 (1.05–1.58)
Cancer mortality			
Monotonical	0.79 (0.64–0.97)	0.85 (0.72–1.00)	0.80 (0.67–0.96)
Female			
All-cause mortality			
≤ 5.5	1.16 (0.95–1.42)	0.93 (0.77–1.13)	0.97 (0.77–1.22)
> 5.5	1.39 (1.24–1.55)	1.24 (1.11–1.37)	1.22 (1.07–1.39)
CVD mortality			
≤ 5.5	1.08 (0.74–1.56)	0.95 (0.68–1.33)	0.94 (0.63–1.39)
> 5.5	1.62 (1.28–2.06)	1.49 (1.13–1.96)	1.62 (1.24–2.13)
Cancer mortality			
≤ 5.5	1.26 (0.88–1.80)	1.02 (0.71–1.45)	1.07 (0.63–1.80)
> 5.5	1.57 (1.18–2.10)	1.72 (1.31–2.28)	1.95 (1.41–2.68)

^a^Model adjusted for age, sex, race/ethnicity, marital status, education level, occupation type, smoking status, alcohol consumption, physical exercise, BMI, eGFR, triglyceride, total cholesterol, fasting glucose, CRP, and ALT

### Results in the obese participants

We then performed the analysis in the obese participants with BMI > 30 kg/m^2^. Similarly, we found a significant association between the SUA levels and the risk of MAFLD in the fully adjusted model (Additional file 1: Table S1). Compared with those in the first group, the ORs (95% CIs) in groups 2–5 were 0.82 (0.47–1.44), 1.50 (0.85–2.66), 1.99 (1.06–3.72), and 2.03 (1.08–3.83), respectively. As for mortality in participants with MAFLD (Additional file 1: Table S2), we observed similar results as the main analysis in the univariate model. When the model was fully adjusted, there was a linear trend for cancer mortality among males [HR (95% CI): 0.62 (0.41–0.94)]. Among females, when SUA > 5.5 mg/dL, elevated SUA levels showed a significantly positive association with risk of CVD mortality [HR (95% CI): 1.59 (1.03–2.44)].

## Discussion

In this study, we found that elevated SUA levels were significantly associated with an increased risk of MAFLD. We also found that SUA levels were non-linear associated with long-term mortality of MAFLD patients. For males, high SUA levels (> 6.7 mg/dL) were associated with an increased risk of CVD mortality, but low SUA levels were associated with an increased risk of cancer mortality. For females, high SUA levels (> 5.5 mg/dL) were associated with increased risk of CVD and cancer mortality.

It is worth noting that adopting the term MAFLD involves not only a change in nomenclature but a more useful criteria in identifying patients with future adverse clinical events [25]. Sirota et al*.* [26] utilized NHANES III data and showed that the crude OR (95% CI) for NAFLD was 1.38 (1.33–1.45) when SUA level was treated as a continuous variable (per 1 mg/dL). In this study, the crude OR (95% CI) of SUA levels for MAFLD was 1.57 (1.48–1.66). This difference may partly reflect a tighter relationship of SUA levels with metabolic disorders. However, after adjusting for metabolic abnormalities and other confounders, the adjusted OR (95% CI) decreased to 1.17 (1.09–1.24). Actually, most of the patients with hyperuricemia have metabolic comorbidities such as diabetes mellitus or metabolic syndrome [27], which may act as both a mediator and a confounder in the relationship where hyperuricemia, metabolic comorbidities and fatty liver each impact the other [28]. Hence, it is difficult to acquire an accurate effect estimate of this relationship simply by the logistic regression model.

The association between SUA and mortality in participants with MAFLD varies across sex and causes in our study. Based on our results, CVD mortality in MAFLD patients was exacerbated by high SUA levels regardless of sex, which was in line with the meta-analysis conducted in the general population [29]. On the contrary, we found that low SUA level was significantly associated with increased cancer mortality in males. This finding was in line with the findings of a large 38-years cohort study that higher SUA levels were associated with a lower risk of lung, colorectal, and prostate cancer mortality among males [30]. Recently, consensus have been reached that controlling SUA levels is beneficial for decreasing cardiovascular risk [31]. However, there is still no clear guide in terms of MAFLD. MAFLD is a phenotype with complex and disparate causes, which indicates that effective treatment will require personalized assessment [1]. Our analyses may offer evidence for the necessity of screening SUA levels in a general population and controlling SUA levels within the appropriate range in MAFLD patients for a better prognosis.

The mechanisms by which uric acid is associated with MAFLD remains unclear, but several studies offered possible explanations. Inflammation has been considered to involve in the pathogenesis of fatty liver disease [32]. Both soluble urate and monosodium urate (MSU) crystal act as a damage-associated molecular pattern, which could trigger inflammatory responses and pathologic consequences [33]. The NOD-like receptor family, pyrin domain containing 3 (NLRP3) inflammasome can be activated by MSU crystals [34], resulting in the production of its downstream effectors interleukin (IL)-1β and IL-18 in gout patients [35]. Correspondingly, our previous study has shown that uric acid regulates hepatic steatosis and impairs insulin sensitivity through activating the NLRP3 inflammasome both in vivo and in vitro [36]. Besides, uric acid acts as a modulator of glucose and lipid metabolism [37]. Choi et al*.* demonstrated that uric acid induces triglyceride accumulation by activating sterol regulatory element-binding protein via induction of endoplasmic reticulum stress in hepatocytes [38]. Furthermore, uric acid generated during fructose metabolism may even amplify the effects of endogenous fructose production and de novo lipogenesis by stimulating aldose reductase in the polyol pathway [39], and inhibiting aconitase in the Krebs cycle [40]. These data may support our findings that uric acid is an important factor in hepatic steatosis.

Moreover, several mechanisms may be responsible for the positive association between SUA levels and CVD mortality in MAFLD patients. It has been reported that uric acid inhibited nitric oxide production, and thereby induced endothelial dysfunction [41]. In addition, hyperuricemia stimulated the vascular renin–angiotensin system, resulting in the excessive production of reactive oxygen species (ROS) [42]. ROS contribute to vascular oxidative stress and endothelial dysfunction, which are associated with the risk of atherosclerosis [43]. Furthermore, higher SUA levels was associated with impaired lipoprotein metabolism, which could induce inflammation in the vessel wall for the development of atherosclerosis [44]. Thus, inflammation processes and oxidative stress were proposed to involve in the connection between uric acid and atherosclerosis, an important player in CVD [45]. Although inducing oxidative stress, uric acid has also been considered as a potentially useful antioxidant, which provides a primary defense against human cancer as a free radical scavenger [46]. Correspondingly, Itahana et al. showed that uric acid transporter SLC2A9, acts as a mediator of the antioxidant function of uric acid (physiological levels) to protect cells from ROS elevation, DNA damage, and cell death. Furthermore, decreased SLC2A9 expression was observed in several cancer types and was associated with a poorer prognosis [47]. Regards to cancer mortality, uric acid is a nutritional marker in hemodialysis patients, which positively associates with laboratory nutritional markers and body composition parameters [48]. A higher SUA levels was strongly associated with a lower risk of all-cause mortality in patients undergoing hemodialysis [48, 49]. Similar to chronic kidney disease, cancer is also a type of nutritional wasting disease. Although MAFLD patients tend to have high SUA levels, we should not ignore the valuable hints of low SUA levels.

We reported for the first time the association of SUA with prevalence and long-term mortality of MAFLD. The advantage of this study is that we used a nationwide, quality-controlled, with a long follow-up period database to draw conclusions. However, there are some limitations in this study. First, NHANES participants are representatives of the US population, therefore the results might not be generalizable to other ethnicities or countries. Prospective studies covering other ethnicities are needed to confirm these study results. Second, although mortality data were longitudinal, factors including SUA levels were available only at baseline. Thus, we could not contain fluctuations during follow-up in our analysis, and it is inevitable to bring selection bias. Furthermore, due to the observational nature of the analysis, we cannot claim direct causality for these results. Third, hepatic steatosis was evaluated by ultrasound without liver biopsy, which is considered as the gold standard. Besides, we are unable to obtain information on the liver cirrhosis of the participants. Fourth, food consumption and medication use that may affect SUA levels were not checked in our study. Those unmeasured variables may potentially influence the relationship between SUA levels and outcomes. Last, specific information about different cancer types is restricted to public. Cancer is a highly heterogeneous disease, so it is interesting to assess the potential function of uric acid in diverse kinds of cancers.

## Conclusion

In this nationwide-based study, we revealed a significant positive association of SUA with prevalence of MAFLD. We also found that elevated SUA levels were associated with increased CVD mortality in MAFLD patients, while low SUA levels were associated with increased cancer mortality in males.

## Supplementary Information

**Additional file 1: Figure S1.** Flow diagram of inclusion criteria from NHANES III. **Table S1.** Association of SUA levels with risk of MAFLD in obese patients. **Table S2**. Threshold effect analysis of SUA on all-cause, CVD, and cancer mortality in obese MAFLD patients.

## Data Availability

The datasets analyzed during the current study are available in the NHANES repository, https://wwwn.cdc.gov/nchs/nhanes/default.aspx.

## Abbreviations

ALT: Alanine aminotransferase
BMI: Body mass index
CVD: Cardio-cerebrovascular disease
CI: Confidence interval
eGFR: Estimated glomerular filtration rate
HR: Hazard ratio
MAFLD: Metabolic dysfunction-associated fatty liver disease
NAFLD: Non-alcoholic fatty liver disease
RCS: Restricted cubic spline
SUA: Serum uric acid
