# Supplementary material for: Association between serum uric acid levels and long-term mortality of metabolic dysfunction-associated fatty liver disease: a nationwide cohort study
Source: Diabetol Metab Syndr. 2023 Feb 23;15:27. doi: 10.1186/s13098-023-00997-z (PMC9948545; doi:10.1186/s13098-023-00997-z)
Supplement: Supplementary file 1 — Additional file 1: Figure S1. Flow diagram of inclusion criteria from NHANES III. Table S1. Association of SUA levels with risk of MAFLD in obese patients. Table S2. Threshold effect analysis of SUA on all-cause, CVD, and cancer mortality in obese MAFLD patients. [file 13098_2023_997_MOESM1_ESM.docx]

**
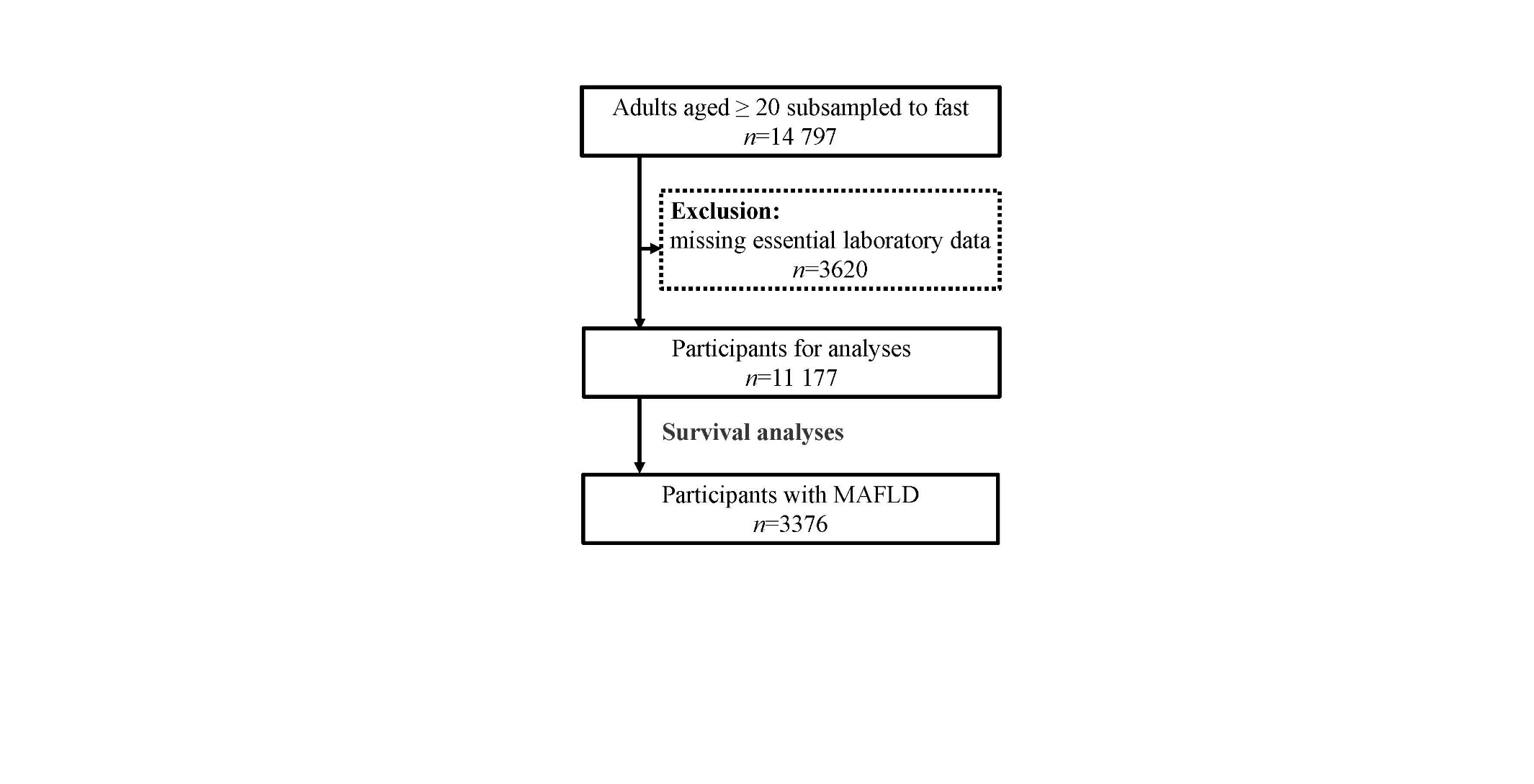
**

**Figure S1** Flow diagram of inclusion criteria from NHANES Ⅲ.

**Table S1. Association of SUA levels with risk of MAFLD in obese patients**

|  | Serum uric acid [OR (95%CI)] | | | | |  |  |
| --- | --- | --- | --- | --- | --- | --- | --- |
| Male | Group 1  (<4.0 mg/dL) | Group 2  (4.0–4.9 mg/dL) | Group 3  (5.0–5.9 mg/dL) | Group 4  (6.0–6.9 mg/dL) | Group 5  (≥7.0 mg/dL) | *P* for trend | Continuous* |
| Model 1 | 1 (ref) | 0.84 (0.52–1.36) | 1.60 (1.00–2.57) | 2.36 (1.37–4.08) | 2.60 (1.56–4.33) | <0.001 | 1.32 (1.20–1.44) |
| Model 2 | 1 (ref) | 0.90 (0.55–1.48) | 1.70 (1.01–2.85) | 2.46 (1.38–4.41) | 2.64 (1.53–4.56) | <0.001 | 1.31 (1.19–1.44) |
| Model 3 | 1 (ref) | 0.82 (0.47–1.44) | 1.50 (0.85–2.66) | 1.99 (1.06–3.72) | 2.03 (1.08–3.83) | <0.001 | 1.24 (1.12–1.37) |

Model 1 was adjusted for demographic factors: age, sex, race/ethnicity, marital status, education level, and occupation type.

Model 2 was adjusted for model 1 plus lifestyle factors: smoking status, alcohol consumption, physical exercise, BMI, and eGFR.

Model 3 was further adjusted for model 2 plus biochemistry factors: triglyceride, total cholesterol, fasting glucose, CRP, and ALT.

*OR for 1mg/dL SUA increase

**Table S2. Threshold effect analysis of SUA on all-cause, CVD, and cancer mortality in obese MAFLD patients**

| Threshold (mg/dL) | HR (95% CI) | | |
| --- | --- | --- | --- |
|  | **Univariate** | **Age-adjusted** | **Multivariate*** |
| Male |  |  |  |
| All-cause mortality |  |  |  |
| ≤6.7 | 0.69 (0.53–0.89) | 0.76 (0.60–0.96) | 0.78 (0.60–1.03) |
| >6.7 | 1.11 (0.83–1.48) | 1.18 (0.99–1.40) | 0.96 (0.77–1.20) |
| CVD mortality |  |  |  |
| ≤6.7 | 0.65 (0.41–1.01) | 0.75 (0.48–1.19) | 0.85 (0.43–1.67) |
| >6.7 | 1.37 (1.03–1.83) | 1.43 (1.18–1.75) | 1.40 (0.95–2.05) |
| Cancer mortality |  |  |  |
| Monotonical | 0.80 (0.65–0.99) | 0.82 (0.64–1.05) | 0.62 (0.41–0.94) |
| Female |  |  |  |
| All-cause mortality |  |  |  |
| ≤5.5 | 0.98 (0.74–1.28) | 0.98 (0.76–1.27) | 0.96 (0.73–1.25) |
| >5.5 | 1.35 (1.16–1.57) | 1.17 (1.01–1.36) | 1.18 (0.95–1.47) |
| CVD mortality |  |  |  |
| ≤5.5 | 0.67 (0.48–0.95) | 0.78 (0.57–1.07) | 0.80 (0.49–1.32) |
| >5.5 | 1.43 (1.03–1.98) | 1.30 (0.92–1.82) | 1.59 (1.03–2.44) |
| Cancer mortality |  |  |  |
| ≤5.5 | 1.29 (0.79–2.12) | 1.15 (0.72–1.83) | 1.08 (0.45–2.60) |
| >5.5 | 1.55 (1.09–2.20) | 1.29 (1.01–1.66) | 1.09 (0.34–3.45) |

* Model adjusted for age, sex, race/ethnicity, marital status, education level, occupation type, smoking status, alcohol consumption, physical exercise, BMI, eGFR, triglyceride, total cholesterol, fasting glucose, CRP, and ALT.
